# EVENTS ASSOCIATED WITH THE OCCURRENCE OF INTRACRANIAL HYPERTENSION IN PEDIATRIC PATIENTS WITH SEVERE CRANIOENCEPHALIC TRAUMA AND MONITORING OF INTRACRANIAL PRESSURE

**DOI:** 10.1590/1984-0462/2020/38/2019123

**Published:** 2020-01-13

**Authors:** Sérgio Diniz Guerra, Alexandre Rodrigues Ferreira

**Affiliations:** a Fundação Hospitalar do Estado de Minas Gerais, Belo Horizonte, MG, Brazil.; b Universidade Federal de Minas Gerais, Belo Horizonte, MG, Brazil.

**Keywords:** Head traumas, Intracranial pressure, Intracranial hypertension, Critical care, Child, Adolescent, Trauma craniano, Pressão intracraniana, Hipertensão intracraniana, Cuidados críticos, Criança, Adolescente

## Abstract

**Objective:**

To determine the events associated with the occurrence of intracranial hypertension (ICH) in pediatric patients with severe cranioencephalic trauma.

**Methods:**

This was a prospective cohort study of patients 18 years old and younger with cranioencephalic trauma, scores below nine on the Glasgow Coma Scale, and intracranial pressure monitoring. They were admitted between September, 2005 and March, 2014 into a Pediatric Intensive Care Unit. ICH was defined as an episode of intracranial pressure above 20 mmHg for more than five minutes that needed treatment.

**Results:**

A total of 198 children and adolescents were included in the study, of which 70.2% were males and there was a median age of nine years old. ICH occurred in 135 (68.2%) patients and maximum intracranial pressure was 36.3 mmHg, with a median of 34 mmHg. A total of 133 (97.8%) patients with ICH received sedation and analgesia for treatment of the condition, 108 (79.4%) received neuromuscular blockers, 7 (5.2%) had cerebrospinal fluid drainage, 105 (77.2%) received mannitol, 96 (70.6%) received hyperventilation, 64 (47.1%) received 3% saline solution, 20 (14.7%) received barbiturates, and 43 (31.9%) underwent a decompressive craniectomy. The events associated with the occurrence of ICH were tomographic findings at the time of admission of diffuse or hemispheric swelling (edema plus engorgement). The odds ratio for ICH in patients with Marshall III (diffuse swelling) tomography was 14 (95%CI 2.8–113; p<0.003), and for those with Marshall IV (hemispherical swelling) was 24.9 (95%CI 2.4–676, p<0.018). Mortality was 22.2%.

**Conclusions:**

Pediatric patients with severe cranioencephalic trauma and tomographic alterations of Marshall III and IV presented a high chance of developing ICH.

## RESUMO

**Objetivo:**

Determinar eventos associados à ocorrência de hipertensão intracraniana (HIC) em pacientes pediátricos com traumatismo cranioencefálico grave.

**Métodos:**

Trata-se de coorte prospectiva de pacientes de até 18 anos, com traumatismo cranioencefálico, pontuação abaixo de nove na Escala de Coma de Glasgow e monitoração da pressão intracraniana, admitidos entre setembro de 2005 e março de 2014 em unidade de terapia intensiva pediátrica. A HIC foi definida como episódio de pressão intracraniana acima de 20 mmHg por mais de cinco minutos e com necessidade de tratamento.

**Resultados:**

Incluídas 198 crianças e adolescentes, 70,2% masculinos, mediana de idade de nove anos. A HIC ocorreu em 135 (68,2%) pacientes; valor máximo de pressão intracraniana de 36,3; mediana 34 mmHg. Receberam sedação e analgesia para tratamento da HIC 133 (97,8%) pacientes, 108 (79,4%) receberam bloqueadores neuromusculares, 7 (5,2%) drenagem de líquor, 105 (77,2%) manitol, 96 (70,6%) hiperventilação, 64 (47,1%) solução salina a 3%, 20 (14,7%) barbitúricos e 43 (31,9%) foram submetidos à craniectomia descompressiva. Os eventos associados à ocorrência de HIC foram os achados tomográficos à admissão de *swelling* (edema mais ingurgitamento) difuso ou hemisférico. A razão de chance para que pacientes com classificação tomográfica Marshall III (*swelling* difuso) apresentassem HIC foi 14 (IC95% 2,8–113; p<0,003) e para aqueles com Marshall IV (hemisférico) foi 24,9 (IC95% 2,4–676; p<0,018). A mortalidade foi de 22,2%.

**Conclusões:**

Pacientes pediátricos com traumatismo cranioencefálico grave e alterações tomográficas tipo Marshall III e IV apresentaram grande chance de desenvolver HIC.

## INTRODUCTION

External causes kill about one million children and adolescents worldwide each year, and among them, traumatic brain injury (TBI) is the leading cause of death, permanent disability, and intensive care hospitalization.^1,2^ Once trauma has occurred, it is up to the health care team to prevent and correct secondary brain damage,^3^ which includes intracranial hypertension (ICH), which may compromise perfusion pressure and brain flow and cause herniations, leading to focal ischemia and brain-stem compression.^4-6^

In the acute phase of trauma, ICH results from swelling (edema and engorgement), hematomas, bruises, edema and, less often, obstructive hydrocephalus. It is worth noting that the changes in intracranial volume and the consequent increase in intracranial pressure (ICP) are the result of complex phenomena that include the intensity of energy transferred at the moment of trauma, whether or not the patient has hypoxia and, probably, unidentified patient-related factors.^5^

Some authors have related the occurrence of ICH to a worse prognosis in adults and children suffering from severe TBI, and reported improved outcomes with aggressive control of ICP.^5,6^ However, the results of studies comparing the outcome of patients who had their treatment guided by ICP monitoring with those who did not are inconclusive.^7-9^ In addition, monitoring has complications such as infections, bleeding, measurement errors and malfunctions, with variable frequency, depending on the device used.^8,10,11^ Reports of prolonged mechanical ventilation, length of stay, unnecessary institution of harmful treatments, and increased hospital costs with and without ICP monitoring are also contradictory. ^8,12,13^

The recommendations for monitoring that are in the “Guidelines for the Clinical Treatment of Severe Head Injury in Infants, Children and Adolescents” are Level III of evidence, “therapeutic option”. ^5^ Determining events associated with the occurrence of ICH in pediatric patients would allow for the identification of those who would benefit from ICP monitoring and those who could be spared the complications and expense of this procedure.

The aim of the present study was to determine the events associated with the occurrence of ICH in pediatric patients suffering from severe TBI with ICP monitoring, in addition to describe the prevalence of ICH, the treatment used, and the group’s outcome regarding death and survival.

## METHOD

This study included a prospective cohort from the period of September 2005 to March 2014. It was conducted at the pediatric intensive care unit (ICU) of the João XXIII Hospital of the Minas Gerais State Hospital Foundation, which is a tertiary public hospital in Brazil, and a reference for emergencies. The hospital predominantly serves pediatric patients who are victims of external causes. Data were obtained by previously trained staff and checked daily by the authors during the study period. The study was submitted and approved by the Research Ethics Committee (Report no. 322/2005).

Patients 18 years of age and younger who were admitted to the pediatric ICU for severe blunt TBI and who underwent ICP monitoring, were included. The following were excluded: patients with gunshot injuries, due to the pathophysiological differences of their injuries, and those whose parents or guardians did not consent to their participation.

Patients in the present study were treated based on the unit’s protocol, which was established in accordance with the Pediatric Guidelines published in Pediatric Critical Care Medicine in 2003 and 2012. ^14,15^ An exception was the criteria for monitoring ICP. The hospital’s neurosurgeons followed the current guidelines for the treatment of adults from the Brain Trauma Foundation. ^16^

ICH was defined as an episode of ICP above 20 mmHg that required treatment, which was performed when the ICP was kept for at least five minutes above this value. This was determined by the intensivist.^15,17^

The severity of TBI in patients aged four years old and older was determined using the Glasgow Coma Scale (GCS). Children below this age were assessed on a scale, and verbal and motor response were adapted for age.^18^ The GCS score was assessed at the time of admission, and six hours after the trauma in order to classify trauma severity. Patients with severe TBI were those with a score below nine in both evaluations. The highest value was used for the purpose of this research. Patients with no motor response, flexion posture or abnormal extension at the time of admission (GCS score of three to five) were grouped for analysis of the association with ICH, because these events have been associated with the occurrence of refractory ICH in previous studies.^11,16^

Cranial computed tomography was performed at the time admission and repeated during treatment as needed. The tomographic findings were gathered into two groups. One with intracranial lesions that had a greater possibility of developing ICH: hemorrhages, bruises, edema, swelling, and the compression or deletion of cisterns. And another with a lower possibility of developing ICH: normal tomography or isolated diagnosis of diffuse axonal lesion.^16^ Marshall tomographic classification was also used to analyze its association with the occurrence of ICH.^19^ The severity of trauma was assessed according to the pediatric trauma score (PTS).^20^

For ICP monitoring, the Codman^®^ catheter was used in the intraparenchymal position and, if it could not be used, intra-ventricular monitoring was performed, or a Richmond screw was installed in the subarachnoid position. Intracranial hemorrhages resulting from ICP monitoring were those that appeared after the device was installed. Patients that needed surgical treatment were analyzed. An analysis of infectious complications resulting from monitoring was not performed due to the difficulty in identifying the cause of the event. Mortality during the patient’s stay in the ICU was described.

The developed database was analyzed in the *Statistical Package for Social Sciences* (SPSS) program version 20.0 (IBM Corp., Armonk, NY, USA). To characterize the groups, we used the quantitative variables of mean ± standard deviation (SD) and median (1st quartile; 3rd quartile) and, for categorical variables, we used absolute frequencies and percentages. Continuous variables without normal distribution were expressed as medians and interquartile range (IQR; 1st quartile and 3rd quartile) and were compared using the nonparametric Mann-Whitney test. Continuous variables with normal distribution were expressed as mean and SD and compared using Student’s *t* test. The comparison of categorical variables was analyzed using the asymptotic Pearson’s chi-square test (when 20% of the expected value was between 1 and 5) and the exact Pearson’s chi-square test (when more than 20% of the expected value was between 1 and 5). Probability was considered to be significant when it was less than 0.05 (p <0.05).

The logistic regression model was adjusted to evaluate the events associated with the occurrence of ICH. Statistical significance was at a level of 0.20. Step by step, the variables with the highest p values were removed until all significant variables remaining at the 0.05 level remained in the final model. The quality of fit was assessed by the Hosmer & Lemeshow test.

The sample size calculation was based on a study of ICU patients between 1998 and 2003, where ICH occurred in 80% of the 134 monitored patients.^11^ Considering the 95% confidence interval (95% CI), the 5% significance level and the 80% study power, the minimum sample size was 110 patients in whom factors associated with the occurrence of ICH were evaluated.

## RESULTS

Between September 2005 and March 2014, 362 patients with blunt severe TBI were admitted to the pediatric ICU, 200 of whom underwent ICP monitoring, and two of whom were excluded from the study because the family did not provide consent. Thus, 198 patients with severe contused TBI and ICP monitoring were included. Of these, 139 (70.2%) were male and their age ranged from three months to 18 years old, with a median age of nine years old (IQ25–75% 5–14 years old).

The types of trauma reported were: 66 (33.3%) patients were run over, 45 (22.7%) received injuries from riding in a car, 30 (15.2%) fell down, 24 (12.2%) received injuries by riding on or driving a motorcycle, 21 (10.6%) received injuries by riding on or driving a bicycle, 6 (3%) were involved in physical altercations and 6 (3%) patients received injuries from some other cause.

The median GCS score at the time of admission was 6 (IQ25–75% 4–7). A total of 71 (35.9%) patients scored between 3 and 5 on the GCS and 127 (64.1%) patients scored between 6 and 8. The PTS score ranged from -3 to 10, with a median of 4 (IQ25–75% 2–5).

The tomographic findings found were: intraparenchymal contusion in 93 (47%) patients, swelling in 87 (43.9%), sub-arachnoid hemorrhage in 77 (38.9%), diffuse axonal injury in 63 (31.8%), subdural hematoma in 57 (28.8%), pneumocephalus in 45 (22.7%), intraventricular hemorrhage in 43 (21.7%), bone collapse in 41 (20.7%), extradural hematoma in 23 (11, 6%) patients, and 8 (4%) presented tomography with no changes for age. The distribution of the Marshall tomographic classifications at the time of admission is found in Table 1. No evacuated expansive lesions were found in any patient.

**Table 1:** Univariate analysis of events associated with the occurrence of intracranial hypertension in 198 patients suffering from severe head injury that needed treatment.

Variable	Total 198 (100%)	Needed treatment (IC) 135 (68.2%)	Did not need treatment (IC) 63 (31.8%)	p-value
Age (years)				
Median (IQ25–75%)	10 (5–14)	9 (5–14)	13 (6–15)	0.22
0 to 1	12 (6.1)	7 (58.3)	5 (41.7)	
2 to 10	80 (40.4)	60 (75)	20 (25)	
11 to 18	106 (53.5)	68 (64.2)	38 (35.8)	
Male	139 (70.2)	94 (67.6)	45 (32.4)	0.22
Type of trauma				
Run over	66 (33.3)	50 (75.8)	16 (24.2)	0.98
Car driver or passenger	45 (22.7)	30 (66.7)	15 (33.3)	
Fall	30 (15.2)	23 (76.7)	7 (23.3)	
Motorcycle driver or passenger	24 (12.2)	10 (41.7)	14 (58.3)	
Bicyclist	21 (10.6)	15 (71.4)	6 (28.6)	
Physical aggression	6 (3)	3 (50)	3 (50)	
Other cause	6 (3)	4 (66.7)	2 (33.3)	
Glasgow				
Median (IQ25–75%)	6 (4–7)	6 (3–7)	6 (5–6)	0.10
3–5	71 (35.9)	54 (76.1)	17 (23.9)	
6–8	127 (64.1)	81 (63.8)	46 (36.2)	
PTS				
Median (IQ25–75%)	4 (2–5)	4 (2–5)	4 (1–5)	0.18
<4	80 (40.4)	49 (61.3)	31 (38.7)	
>4	114 (57.6)	80 (70.2)	34 (29.8)	
Marshall				
I	11 (5.6)	4 (36.4)	7 (63.6)	<0.0001
II	83 (41.9)	48 (57.8)	35 (42.2)	
III	67 (33.8)	56 (83.6)	11 (16.4)	
IV	10 (5.1)	9 (90)	1 (10)	
NEML	27 (13.6)	16 (59.3)	11 (40.7)	

ICH: intracranial hypertension; IQ: interquartile range; PTS: pediatric trauma score; NEML: non-evacuated mass lesion.

ICH occurred in 135 patients (68.2%) and the maximum ICP value was a median of 34 mmHg (IQ25–75% 22.5–45 mmHg). Patients required some treatment for ICH with a median of 3.2 days (IQ25–75% 1–5 days), and they remained on ICP monitoring for an average of 3.2 ± 2.3 days.

Regarding the treatment for ICH, 133 (97.8%) required sedation and analgesia for treatment, 108 (79.4%) required neuromuscular blockers, 7 (5.2%) needed CSF drainage, 105 (77.2%) needed mannitol, 96 (70.6%) needed hyperventilation, 64 (47.1%) needed 3% saline, 20 (14.7%) required barbiturates and 43 (31.9%) needed a decompressive craniectomy.

The intraparenchymal Codman^®^ catheter was used for ICP monitoring in 145 (73.2%) patients. Seven (3.5%) patients used intraventricular monitoring and, in the early years of the study, due to the large volume of patients in the hospital, 46 (23.2%) used a Richmond screw in the subarachnoid position. Nine (4.5%) patients had secondary hemorrhaging when the ICP monitoring device was installed. None of them requiring surgical intervention. The monitoring device malfunctioned in 24 patients (12.1%), requiring 9 of them to be replaced.

Of the 198 patients included, 44 died (22.2% mortality). Of these 44 patients, 38 had ICH (86.4%) and 6 (13.6%) did not (chi-square; p = 0.007).

Univariate analysis of the events associated with the occurrence of ICH is described in Table 1. The distribution of maximum ICP values according to the Marshall classification was: Marshall I, median 19.5 mmHg (IQ25–75% 15–27.5); Marshall II, median 28 mmHg (IQ25–75% 21–40); Marshall III, median 35 mmHg (IQ25–75% 15–27.5); Marshall IV, median 43 mmHg (IQ25–75% 24–52); non-evacuated expansive lesion, median 37.5 mmHg (IQ25–75% 18–46).

Table 2 relates the results of the final multivariate analysis model to the variables that were statistically significant. Patients with a Marshall III tomographic classification were 14 times more likely to have ICH, and those with a Marshall IV tomographic classification were 24.9 times more likely.

**Table 2:** Multiple analysis of the events associated with the occurrence of intracranial hypertension in 198 patients suffering from severe head injury that needed treatment.

Variable	*Odds Ratio*	95%CI	p-value
Marshall III	14	2.8–113	0.003
Marshall IV	24.9	2.35–676	0.018
Glasgow	2.13	0.88–97.85	0.186
Pediatric trauma score	1.183	0.781–108.6	0.260

95%CI: 95% confidence interval.

## DISCUSSION

The case series presented is relevant, considering that the average number of US hospitals with the highest ICP monitoring volume is 11 pediatric patients per year.^21^The percentage of patients undergoing monitoring classifies the Brazilian hospital as an “aggressive center”, as reports from the United Kingdom and the United States show monitoring percentages between 7.7 and 59%.^22,23^ Stein reports improved outcomes of patients treated at centers with aggressive monitoring and treatment.^24^

The predominance of male patients, aged between nine and ten years old and victims of traffic accidents, is in agreement with previous reports from Mexico, Brazil and South Africa.^10,11,25^ The GCS score distribution at the time of admission and the PTS classifications show that this was a group of critically ill patients. Furthermore, the tomographic description that shows multiple injuries per individual reinforces the impression of the complexity of the trauma suffered.

The small number of patients that had a normal tomography or had an isolated CT diagnosis of diffuse axonal injury with ICP monitoring, suggest that most neurosurgeons followed the Brain Trauma Foundation recommendations for adults.^16^ Recommendations for adults during the study period included ICP monitoring in patients with severe TBI and altered tomography or in patients with severe TBI and normal tomography, if two or more of the situations - such as an age over 40 years old, unilateral or bilateral abnormal motor posture and systolic blood pressure <90 mmHg - were observed upon admission to the hospital.^16^ On the other hand, pediatric consensus recommended that monitoring could be considered in children with severe TBI regardless of the tomographic findings.^14,15^

The distribution of measurement frequency for the treatment of ICH suggests that the sequence proposed by pediatric guides was followed in most of the cases, from the least aggressive to the most aggressive, according to the characteristics of the patients, the lesions, and the response to the instituted treatment.^5,14,15^

Monitoring of bleeding complications occurred in a small percentage of patients and did not represent serious events. Other studies have shown similar results.^8,11^ Perhaps the most common complication was the maintenance of aggressive monitoring and treatment in patients who did not need it.

PTS also did not correlate with the elevation in ICP, as reported by Figaji et al. However, the author found a correlation with the pediatric mortality rate of -2, which is a good parameter to be analyzed in future studies.^26^

In the present study, patients were divided into groups with scores of three to five and six to eight in the GCS. There was no difference in the occurrence of ICH between the groups, unlike a previous study with pediatric patients with severe TBI, in which the presence of abnormal positions at the time of admission correlated with the occurrence of refractory ICH.^11^

The present study showed an association between Marshall CT classification in III and IV and the occurrence of ICH in pediatric patients with severe TBI through a multivariate analysis. This is a finding that has a practical application and, moreover, shows the relevance of using the Marshall classification for pediatric patients, even though its midline deviation and mass lesion values were stipulated based on the size of an adult skull.^19^ The data suggest that pediatric patients who are in a Marshall III or IV tomographic coma should be monitored or treated aggressively based on clinical and tomographic data, where monitoring is not available. Unmonitored treatment is acceptable, as there is no evidence in the literature that ICH treatment guided by ICP monitoring generally improves outcomes, but it is not recommended for patients at a high risk of refractory ICH and those who need a craniectomy.^7,8,27,28^

The study was designed and executed to meet the proposed objectives of identifying events associated with the occurrence of ICH in children and adolescents with severe TBI and with ICP monitoring. The calculated sampling was achieved and, because the occurrence of ICH was within the expected range, the results were reliable. However, a limitation of the study was that the analysis of the association of systemic arterial hypotension with an occurrence of ICH, as already described for adults, was not included. This factor was not evaluated in the study due to the limitations of data collection during follow-up. Other limitations that may be cited were the long amount time it took to collect the data, the lack of monitoring of the complications associated with ICH monitoring, such as infections, and the use of different ICP monitoring devices in a smaller range of patients.

The percentage of deaths among patients who had ICH was three times higher than among those who did not, which confirms the findings of other authors regarding the relevance of this cause of brain damage.^4,5^ The 22% mortality result found in the present study is in line with expectations for reference centers, which is slightly above 20%. ^24,29^

It can be concluded that pediatric patients with severe TBI and Marshall III and IV tomographic alterations had a high chance of developing ICH, suggesting that this is a parameter that can indicate the need for ICP monitoring.
